# Prediction of Chromatography Conditions for Purification in Organic Synthesis Using Deep Learning

**DOI:** 10.3390/molecules26092474

**Published:** 2021-04-23

**Authors:** Mantas Vaškevičius, Jurgita Kapočiūtė-Dzikienė, Liudas Šlepikas

**Affiliations:** 1 Department of Applied Informatics, Vytautas Magnus University, LT-44404 Kaunas, Lithuania; jurgita.kapociute-dzikiene@vdu.lt; 2 JSC Synhet, Biržų Str. 6, LT-44139 Kaunas, Lithuania; liudas@synhet.com

**Keywords:** deep learning, chromatography, neural networks, machine learning, solvent prediction, organic synthesis, purification

## Abstract

In this research, a process for developing normal-phase liquid chromatography solvent systems has been proposed. In contrast to the development of conditions via thin-layer chromatography (TLC), this process is based on the architecture of two hierarchically connected neural network-based components. Using a large database of reaction procedures allows those two components to perform an essential role in the machine-learning-based prediction of chromatographic purification conditions, i.e., solvents and the ratio between solvents. In our paper, we build two datasets and test various molecular vectorization approaches, such as extended-connectivity fingerprints, learned embedding, and auto-encoders along with different types of deep neural networks to demonstrate a novel method for modeling chromatographic solvent systems employing two neural networks in sequence. Afterward, we present our findings and provide insights on the most effective methods for solving prediction tasks. Our approach results in a system of two neural networks with long short-term memory (LSTM)-based auto-encoders, where the first predicts solvent labels (by reaching the classification accuracy of 0.950 ± 0.001) and in the case of two solvents, the second one predicts the ratio between two solvents (R^2^ metric equal to 0.982 ± 0.001). Our approach can be used as a guidance instrument in laboratories to accelerate scouting for suitable chromatography conditions.

## 1. Introduction

Cheminformatics is a rapidly evolving field, especially with modern machine-learning algorithms. Big data and GPU-accelerated algorithms present solutions to routine problems faced by organic chemistry specialists. Organic chemistry is beneficial for such fields as medicine, biochemistry, biotechnology, pharmaceutical, agrichemical, and others, constantly shaping human life and our society [1]. In practice, chemistry presents unique challenges due to the enormous chemical space size of synthetically feasible molecules (<500 Da) that is [2,3]. Various approaches have been offered to tackle challenging problems (such as optimization of chemical reactions, retrosynthesis, or drug design). However, outdated rule-based prediction algorithms [4,5] have been recently replaced with machine learning [6], as in [7,8,9,10]. Rapid and lower-cost production of new synthetic molecules has recently received a lot of interest from the chemists’ research community, especially with the advent of AI-assisted drug discovery [11,12]. However, a significant bottleneck for the production of novel compounds is purification, which can be accelerated with AI-based solutions. In this paper, we focus on the laboratory technique, namely chromatography which is used to purify compounds by separating a chemical mixture. Although this method is widely used in organic synthesis, its drawback is the need to identify appropriate conditions: solvents and the ratio between solvents. Traditionally, the process of scouting suitable chromatography conditions is performed by manually testing series of solvent combinations and ratios between them using thin-layer chromatography (TLC) as the primary tool. The objective of this paper is to develop an effective methodology based on modern machine-learning algorithms for modeling of solvent systems and ratio between solvents used for chromatographic purification. Two novel datasets were created for this task that contain a wide variety of reaction types and are not related to a limited range of compounds. In our paper, we test three vectorization methods (extended-connectivity fingerprints, learned embedding, auto-encoders) and three types of deep neural networks (feed-forward neural network (FFNN), convolutional neural networks (CNN), long short-term memory (LSTM)). The main advantage of such a tool is the ability to facilitate the scouting of acceptable purification conditions that may lead to higher throughput in laboratories. Also, fewer solvents and materials could be used when manually scouting using TLC. Our research is based on the premise that the prediction of chromatography conditions can be transferred from the artificial environment to the real with deep neural networks (DNNs). Similar premises have been investigated, e.g., prediction of the retention time for the peptide chromatography with DNNs [13,14] have already led to very successful results. Prediction tasks are only accurate if machine learning is performed in a supervised manner and only if enough quantity and quality of training data are available. Big noise-free and diverse data is needed to reflect various experimental conditions and assure high accuracy levels; unfortunately, such datasets are often proprietary or do not exist.

## 2. Related Work

In our paper, we focus on the purification of final products because it is an essential element of successful synthesis and ubiquitous to various industries. Traditional methods for liquid separation (such as liquid/liquid extraction) have some disadvantages: low efficiency and flux increase time and cost of separation [15]. A mixture separation technique known commonly as column chromatography is often used [16]. The method is useful for the purification of various mixtures of organic synthesis [17].

To facilitate the process of chromatographic purification, various approaches for the prediction of retention time have been used, ranging from simple log-P-based models [18] to more complex models based on artificial neural networks [19]. Retention time modeling is one of the methods for scouting suitable chromatographic conditions by predicting how much time is taken for a compound to pass through the chromatography column. Deep learning has been used to predict the retention time of peptides [13] and steroids [20]. Other machine-learning methods such as multiple linear regression [21], partial least squares regression [22], support vector machine [23] also have been tested and achieved a considerable level of accuracy. A distinction between global and local modeling is important and has been clarified in a review paper [24]. Local modeling can relate to a limited range of solvent compositions or to a limited range of analytes for which the model is suited. Global models that can accurately model a wide range of solvent compositions and analytes are generally more useful; however, they require more data and are more complex. Direct prediction of solvent systems suitable for chromatography conditions is a different method of modeling chromatographic techniques. Multiple linear regression algorithm has been used for prediction of the solvent system of counter current chromatography and was found accurate for 9 out of 21 bi-phasic solvent systems [25].

In recent years, deep learning has emerged as one of the effective supervised machine-learning solutions with a wide variety of applications. Chemical compounds are vectorized to become a suitable input for the machine-learning algorithms to process. Various molecular representation methods have been implemented, simpler methods such as one-hot encoding [26,27] or vectorization of bond strings [28]. Molecular descriptors have also been shown to be useful for the representation of compounds [29,30]. More complex methods used with ANNs have been implemented to represent structures in latent space with the use of auto-encoders [31], variational encoders [32], conditional variational auto-encoders [33], and other unique methods such as Attentive fingerprints (FP) that uses a graph attention mechanism [34]. However, it is important to notice that there is no consensus on which vectorization method is the best: different methods are the best for different tasks. Morgan fingerprints or extended-connectivity fingerprints (ECFP) [35] have been used to vectorize molecules which then are used with deep neural networks to predict reaction outcomes [36]. The long short-term memory (LSTM) method with memory and feedback connections between cells is suitable for training on sequences and therefore used in a sequence to sequence (seq 2 seq) approaches for chemical synthesis pathway design [37]. In addition, convolutional neural networks (CNNs) were successfully used to evaluate chemical molecules by first producing a digital image of the structure and later feeding it to the neural network to classify it [38]. Similar to vectorization methods, the optimal types and topologies of neural networks used in the field of organic synthesis are very dependent on the task.

In summary, modeling of chromatographic conditions is usually done by predicting the retention time of the compounds within a mixture. However, it is essential that the structure of molecular impurities is known, and additional computation is required to produce a specific solvent system for purification. However, organic synthesis reaction mixtures more than often include by-products that are formed during synthesis which cannot be directly identified. Therefore, a system that can produce predictions for chromatography conditions must at least to some degree intrinsically approximate possible by-products in the reaction mixture. D. M. Lowe’s dataset [39] used in our research is extracted from US patents contains data about organic synthesis reaction compounds and chromatographic conditions used for purification of synthesis mixtures. We have used the unstructured text data to build two new datasets for modeling of solvent system and the ratio of solvents used for chromatographic purification. In our paper, we test three vectorization types along with different types with neural network modification to demonstrate a novel method for modeling chromatographic solvent systems employing two neural networks in sequence. Since our system’s input is compounds of the reaction, not the compounds of the resultant mixture, a difficult step of identifying impurities in the laboratory is not required. This unique feature provides an opportunity to directly predict the solvents and the ratio between solvent used in the process of chromatographic purification, which can facilitate the scouting of chromatographic conditions.

## 3. Formal Definition of Tasks

In this research paper, we are solving two tasks:

- Task No. 1: Prediction of the solvent labels that make up the solvent system for chromatographic purification.
- Task No. 2: Prediction of the ratio between solvents if two solvents are used.

These tasks can be formally defined as follows:

**Task No. 1 (Multiclass Classification)****Task No. 2 (Regression)**InputLet a chemical reaction be denoted as *d_i_* and belong to a space of chemical reactions *d_i_* ∈ *D*. Each *d_i_* can be converted into a p-dimensional feature vector *X_i_* = (*x_i_*_,1_, *x*_i,2_, …, *x_i,p_*)OutputLet *Y =* {*y*_1_*, y*_2_*, … y_N_*} be a label space of size *N* that represents possible solvents (class) labelsLet *γ_i_* ⊆ [0, 1] be a continuous variable which represents the ratio between the solventsPrediction functionLet *η* be a function that *η*(*X*) → *Y* correctly predictsa set of solvents labelsa ratio between solventsMethodLet *Γ* be a machine-learning algorithm that finds an approximation *η*′ (model) of a function *η* when given a learning dataset *D^L^* ⊂ *D*


Both tasks aim to find the closest approximation (a model) *η*′ of function *η* that produces the most accurate predictions on the testing dataset *D^T^* ⊂ *D*. The training *D^L^* and testing *D^T^* datasets are not overlapping in our experiments (*D^L^* ∩ *D^T^* = ∅) and *D^T^* is composed to have enough diversity of chemical reactions and their correct distribution in the space. Due to both reasons, we can assume that the evaluation results on *D^T^* will indicate how accurate the model is for unseen instances: i.e., on *D*–*D^L^*–*D^T^*.

## 4. The Data

The dataset for deep learning has been created using Daniel Mark Lowe’s and NextMove’s open-source collection of chemical reactions extracted from US patents issued from 1976–2016 [39]. It is composed of 3.7 million reactions in simplified molecular-input line-entry system (SMILES) notation, synthesis descriptions, and additional information. Synthesis description is a paragraph of text that specifies what actions are taken to perform the synthesis. The dataset is a structured XML file, whereby all synthesis instructions are expanded into separate actions by segmentation of relevant parts of the paragraph that relate to the action. Employing this structure, we have been able to use the data of a particular synthesis step, so-called purification to form datasets, whereby an object—a chemical reaction is described by solvents (nominal labels) used in the purification step and by the ratio between solvents. Only reactions that have employed normal-phase chromatographic purification have been used in our experiments. Table 1 illustrates a few examples of the synthesis instruction paragraph segment (purification) in the raw text column. Other columns Solvents and Ratio present the extracted labels and the ratio between solvents, respectively. One or two solvents in the purification step are commonly used in reactions (which represents 95.57% of all cases in our dataset). The rest of the cases (with three or more solvents) are too rare to form a sufficient subset for training a machine-learning algorithm. The required data extraction was performed by a custom-made script that first compiled a list of all used solvents in the chromatographic purification and then automatically matched it to the raw text.

**Figure 1:**
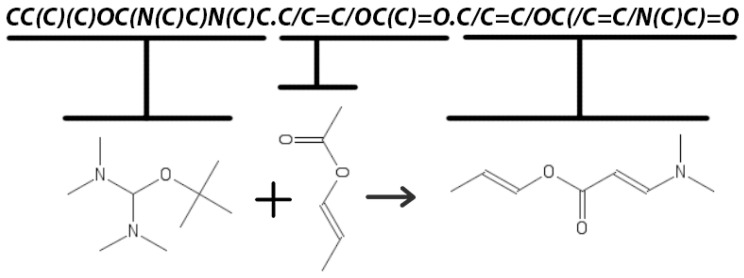
A graphical representation of the reaction and its notation in SMILES. This relation maps SMILES encoding segments into corresponding molecules.

All instances in datasets are chemical reactions expressed with sequences of symbols representing reactants’ chemical structure and reaction products. The simplified molecular-input line-entry system (SMILES) [40] allows for the graph-like structure of a molecule to be written in one line of symbols. Each individual molecule is separated by “.” a dot symbol. Figure 1 illustrates an example of a chemical reaction with a graphical picture of molecules and how it appears in the datasets as a symbol sequence that denotes three molecules separated by a dot. Three molecules: reactant 1-*CC(C)(C)OC(N(C)C)N(C)C*, reactant 2-*C/C = C/OC(C) = O*, product-*C/C = C/OC(/C = C/N(C)C) = O* are separated by a *“.”* dot symbol.

Two datasets named DS1 and DS2 were created for tasks No. 1 and No. 2 (described in Section 3), respectively. The necessity of two datasets (created from the original one) is based on the fact that we are solving two different tasks that have different outputs. Only labels of solvents and ratios between solvents are needed, but other contextual text information is irrelevant to the solving tasks. Thus, the DS1 dataset is used to train a model for the prediction of solvents used in chromatographic purification (corresponds to task No. 1 in Section 3), while DS2 is used to train a model for the prediction of the ratio between solvents (task No. 2 in Section 3). Datasets are described in more detail in this section below.

Table 2 contains snippets from datasets DS1 and DS2 (their inputs and outputs). DS1 and DS2 both have a sequence of molecules in SMILES notation as inputs. DS1 has solvent labels as outputs. DS2 has a single continuous output variable that describes the ratio between the solvents. The ratio is normalized by dividing each number by a sum of both. For instance, the ratio of 9:1 is scaled to 0.9:0.1. A sum of both normalized values is always equal to 1 (0.9 + 0.1 = 1), which means that only one value needs to be predicted, (0.9) and the other is calculated (1 − 0.9 = 0.1). Binary labels have only one format. For example, only ethyl acetate and hexane exist while hexane and ethyl acetate does not. Therefore, when a solvent system of ethyl acetate and hexane is predicted with the ratio of 0.9, it indicates the value for the first label as they appear in the dataset. Ethyl acetate would be 0.9 and hexane 0.1 (1 − 0.9 = 0.1). This makes the output value of the ratio a single continuous value and reduces the complexity to achieve higher accuracy. In essence, the prediction of both ratio components would be redundant and could lead to a more complex regression task. Hence, the normalization reduces the calculation complexity, makes the output more suitable and stable for supervised machine-learning algorithms, but at the same time does not distort the data.

In total, the dataset DS1 consists of 454,259 instances (*d_i_*), with a split of 10% as the testing dataset (45,426 instances) and 90% as the training (408,833). Each instance has either one or two solvent labels (~1.8 labels on average per instance). DS1 contains ten labels (acetone, ethanol, hexane, toluene, petroleum ether, methanol, chloroform, ethyl acetate, dichloromethane, diethyl ether) for solvents. In total, there are 52 possible unique combinations: 10 single labels and 42 two label sets. The dataset is imbalanced and the top 5 most common label sets are ethyl acetate and hexane, ethyl acetate, methanol and dichloromethane, dichloromethane, chloroform, and methanol. Table 3 represents the distribution of instances over different class labels of the training and testing subsets in DS1.

The trained models are only considered reasonable if the testing dataset’s accuracy exceeds random (Equation (1)) and majority (Equation (2)) baselines. The random baseline naively determines the accuracy boundary based on probabilities of classes Yi in the dataset. The majority baseline determines the accuracy when all instances are attached to the major class *Y*_top_.

(1) $$\mathrm{Random}\;\mathrm{baseline}=\sum_{i=1}^{n}\left(P{\left(Y_{i}\right)}^{2}\right)$$

*n*—dataset size, (*P*(*Y_i_*))—the probability of *Y_i_* class.

(2) $$\mathrm{Majority}\;\mathrm{baseline}=\max\left(P\left(Y_{\mathrm{top}}\right)\right)$$

Random and majority baselines for the DS1 dataset are equal to 0.172 and 0.313, respectively. Since the majority baseline is higher (compared to random), the goal of our research is to exceed it.

The dataset DS2 consists of 272,329 number of instances (*d_i_*), with a split of 10% as the testing dataset (27,233 instances) and 90% of the training dataset (245,096 instances). Dataset DS2 is smaller because it is only used to predict the ratio for binary systems. DS1 contains examples that have one or two solvent labels. In the case of a single solvent systems, ratio is not needed. The total number of instances of DS1—454,259. The number of one solvent systems in DS1—180,669. This leaves: 454,259–180,669 = 273,590 binary systems. DS2 contains the ratio of the binary solvents (272,329). The number on binary systems (around 1000) was not used due to inaccurate extraction from the original dataset, for example, the ratio being 0:1 or 0:0). Table 4 presents descriptive statistics about the dataset DS2. Because the target value of task No. 2 is a float variable, the dataset is described using different statistical characteristics such as mean, standard deviation (Std), minimum (Min), and maximum (Max) values. In addition, 25%, 50% and 75% quartiles are included. Values of the same metrics are similar for training and testing subsets, which assures similar training/testing conditions.

For task No. 2, regression problem, two baseline metrics were calculated using central tendency measures, mean baseline, and median baseline. Baseline metrics have been evaluated by taking mean and median separately of the testing dataset and calculating MSE with the testing dataset using formulas (Equations (3) and (4)) where $\bar{y}$−mean, $\tilde{y}$−median. In contrast to task No. 1, the goal is to create models with lower MSE scores than mean and median baselines.

(3) $$\mathrm{Mean}\;\mathrm{MSE}\;\mathrm{baseline}=\frac{1}{n}\sum_{i=1}^{n}{\left(\bar{y}-y_{i}\right)}^{2}$$

(4) $$\mathrm{Median}\;\mathrm{MSE}\;\mathrm{baseline}=\frac{1}{n}\sum_{i=1}^{n}{\left(\tilde{y}-y_{i}\right)}^{2}$$

Mean MSE baseline = 0.094, Median MSE baseline = 0.105.

Despite all reactants and products of the reaction being combined into a sequence, the neural network model must be independent of all possible permutations. For example, the order of *reactant 1, reactant 2, reactant 3*, can be changed to *reactant 2, reactant 1, reactant 3*, etc. Since it is entirely unclear in what order separate molecules will be presented to the models, they must be prepared to predict correctly in any case. For this reason, created datasets were augmented with the new instances by permuting sub-strings (molecules) for all objects in the dataset. To control the exponential growth of new instances, we have restricted the maximum permutation number per instance to the range of [2,3,4,5,6,7]. After additional analysis, this value was set to 5. The lower values could not sufficiently cover typical forms of inputs, whereas the higher values generated redundant instances. Redundant training instances usually lead to overfitting and a decrease in the model’s accuracy.

## 5. Materials and Methods

### 5.1. Vectorization

**Figure A1:**
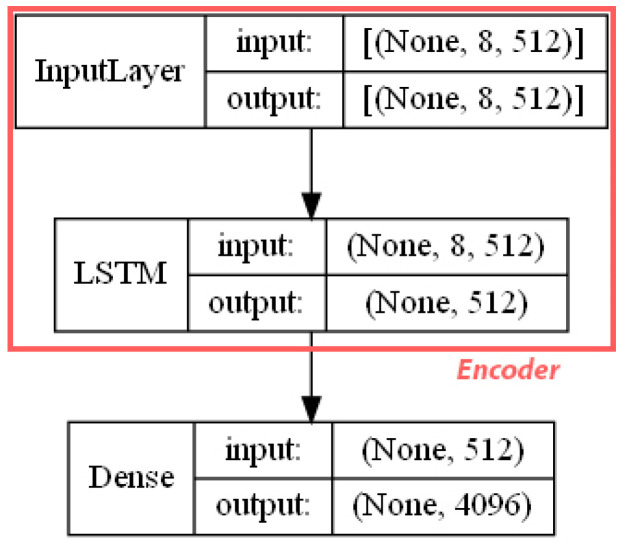
The feed-forward auto-encoder.

**Figure A2:**
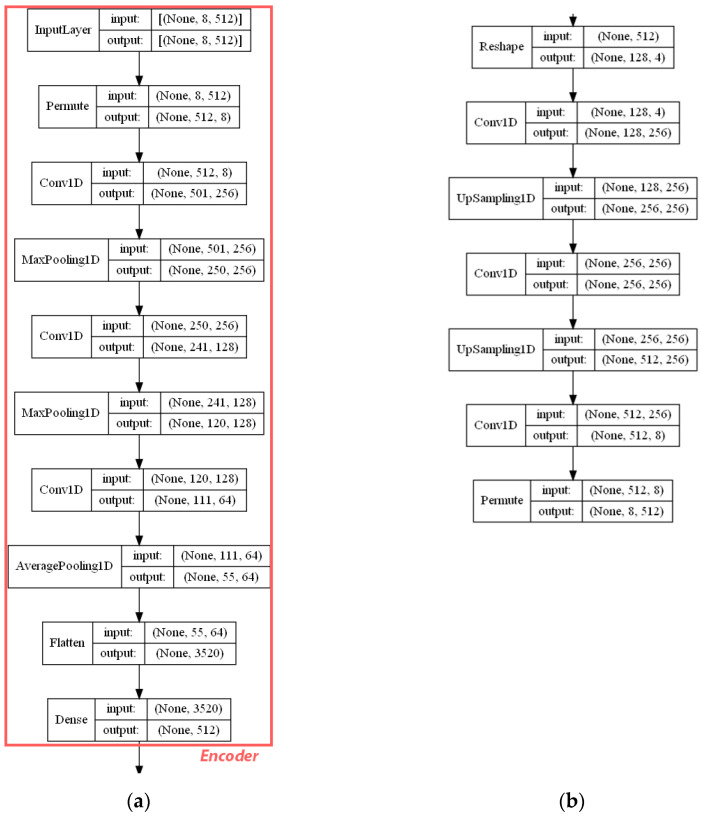
The 1D CNN auto-encoder composed of encoder (**a**) and decoder (**b**).

**Figure A3:**
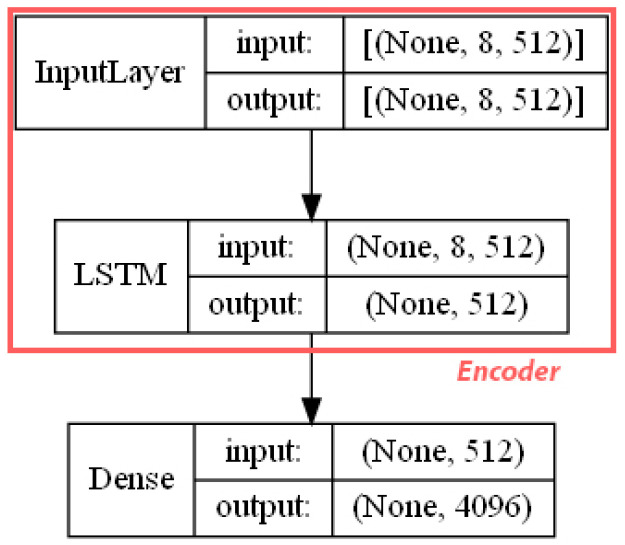
The LSTM auto-encoder.

**Figure A4:**
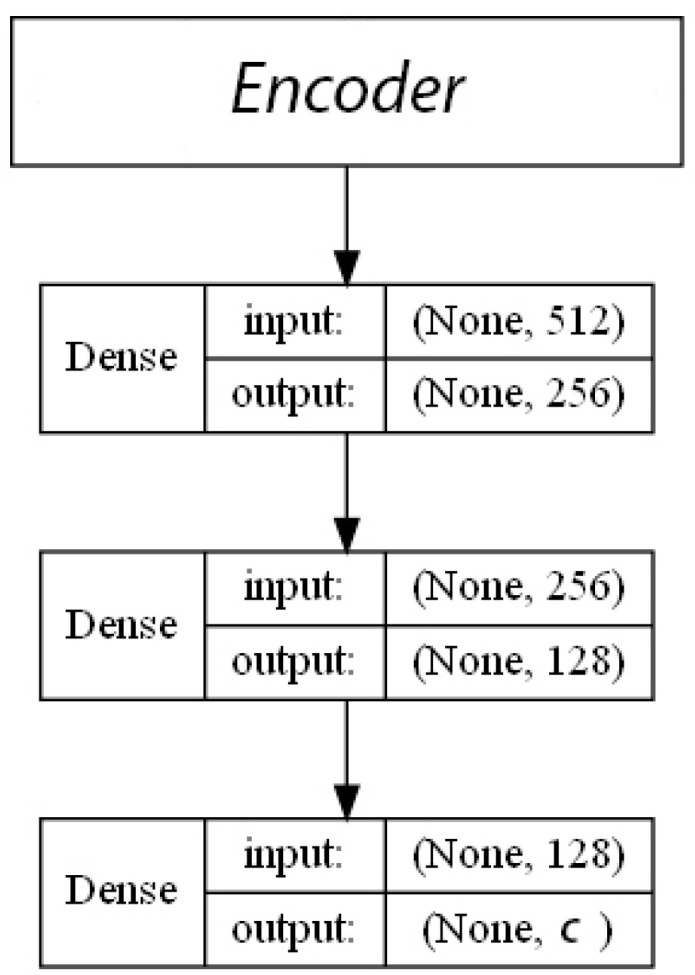
The optimal determined topology and hyper-parameters of a simple FFNN in combination with encoders. The notation c was set to 10 and 1 in tasks No. 1 and No. 2, respectively.

For supervised machine-learning algorithms, the input data must be a matrix or a vector containing numeric values. In our case, the symbolic line representing chemical structure in SMILES notation must also be transformed into a form compatible with the input layer of a DNN. Our investigated embedding types:

- Learned Embedding (LE). An object’s attributes—chemical structures in SMILES notation are concatenated into one line of symbols with a dot as their separator. The downstream supervised machine-learning task symbol representations (embeddings) can be learned jointly. 56 unique symbols are used in the SMILES representation and they all must be represented with N-dimensional vectors. N was set to 12 because larger values did not increase the accuracy in the preliminary experiments. Not to lose the input information, the maximum length of a symbol sequence was set to 200. Thus, the embedded 2D vector for any input sequence consisted of 200 rows (one row per symbol) of 12 vector values each. In short, each of 56 unique symbols is embedded with vectors of 12 dimensions that are trained the same way as weights are trained in a neural network. For a single instance, a matrix 200 × 12 is formed by looking up the symbol and its vector and later combining them into a matrix [41]. Some key advantages of a learned embedding type are the following: (1) encoding is fast, as it does not require any additional processing if compared to, e.g., fingerprints or descriptors; (2) embedding vectors are jointly learned with the training model, therefore complements each other well; (3) compared to one-hot encoding, learned embeddings provide a more uniform and less sparse vectorization which may extract more features of the molecular compounds.
- Extended-connectivity fingerprints (ECFP). Morgan fingerprints or extended-connectivity fingerprints (ECFP) [42] are representations of molecular structures explicitly created to capture chemical features. ECFPs are a variant of the Morgan algorithm that solves the molecular isomorphism problem, where two molecules numbered differently should produce the same fingerprint vector. ECFPs are very useful for the representation of topological structural information and have been used in a diverse set of applications, such as virtual screening [42], activity modeling [43], and machine learning [44,45,46]. Since the maximum number of molecules participating in a single reaction is 8, it results in an 8 × 512 matrix composed for every reaction. The main features of ECFPs are that they represent the presence of particular substructures by means of circular atom neighborhoods. ECFPs represent both the presence and absence of functionality, which are significant for extracting the molecule’s features. This results in a more informative vectorization method. Compared to the one-hot encoding method able to encode only characters or symbols, ECFPs encode fragments of the molecular structure.
- ECFP auto-encoder (ECFP + E). Auto-encoders have been offered as the dimensionality reduction solution for rather large and sparse matrices of extended-connectivity fingerprints. The auto-encoder consists of two connected neural networks called encoder and decoder. Training of a neural network works by taking in ECFPs and trying to reproduce the same fingerprints using a bottleneck layer called a latent space. Later, encoder weights are transferred into a separate neural network. During the training, each instance passes the encoder layer and is compressed to the latent layer’s size. It is even hypothesized that an auto-encoder can denoise the data by learning only the key features representing a specific reaction. The main advantage of an encoder is that it learns how to map the larger multiple molecules input into smaller vectors during the training phase. This action forces the encoder to detect relevant ECFP parts and disregard irrelevant, which, in turn, may lead to higher accuracy. Auto-encoders were also proved to be successful for the dimensionality reduction in the QSAR (Quantitative structure-activity relationship) modeling [47].
  Furthermore, we present approaches (including encoder-decoder parts, topologies, and hyperparameter values) used to develop auto-encoders for vectorization.
  Three types of auto-encoders were constructed and investigated: (1) feed-forward, (3) 1DCNN, and (2) LSTM. Two types of input formats were used: (1) an unchanged ECFP with a size of 8 × 512 for LSTM and 1DCNN auto-encoders; (2) a flattened ECFP of size 1 × 4096 with a feed-forward auto-encoder for feed-forward. These input types were used to simplify encoders’ topology: i.e., the feed-forward auto-encoder does not require an additional flattening layer if the input is a one-dimensional vector.
  The latent dimension size, which is the key auto-encoder’s parameter, was set to 512. Out of tested latent dimensionality sizes (32, 64, 128, 256, 512) in the preliminary experiments, 512 produced the most accurate reconstructions and at the same time assured significant compression by 8 times compared to the vector size (4096) of the initial input. Binary cross-entropy loss function was used for the training of auto-encoders.
  Topologies of auto-encoders were selected experimentally based on the lowest loss scores on the testing dataset. These experiments helped us to end up with an exact parameter set for this particular vectorization type. The tuning of parameters was performed separately with each classification method. The optimization of parameters was performed by testing all values of the selected parameter, setting the best determined one, and then iteratively advancing to the optimization of the following parameter. Deep auto-encoders have been constructed by stacking feed-forward layers with different numbers of neurons (16, 32, 64, 128, 256, or 512). However, these deeper architectures underperformed shallow auto-encoders, therefore, were not selected for our further experiments. The investigation of the 1DCNN auto-encoder included the addition/removal of convolutional layers (with sizes of 8, 16, 32, 64, 128, 256), their kernels (with sizes from 2 to 20), and pooling layers (with sizes of 2, 3, 4, 5). The experiment investigating revealed the optimal topology containing three convolutional layers of sizes (256, 128, 64 in sequence with kernel sizes of 12, 10, 10) and pooling layers of sizes (2, 2, 2). Various LSTM auto-encoders were tested by stacking LSTM layers with different numbers of neurons (16, 32, 64, 128, 256, or 512). However, deeper architectures underperformed shallow auto-encoders.
  Figure A1, Figure A2 and Figure A3 (Appendix A) illustrate the topologies and sizes of layers of all three previously described auto-encoders (Feed-forward, LSTM, and 1D CNN). Figure A1 (Appendix A) represents feed-forward auto-encoder with a flattened input of 4096, one latent layer size of 512; Figure A2 (Appendix A) represents a 1D CNN auto-encoder. Kernel sizes of encoder are 12, 10, 10; kernel sizes of decoder are 1, 1, 1, 1; rectified linear units (ReLU) activation function used for convolutional layers. Figure A3 (Appendix A) represents the LSTM auto-encoder with one LSTM layer of size 512.
  Encoders were later combined with a simple feed-forward neural network (FFNN) illustrated in Figure A4 (Appendix A). All encoders output a 1 × 512 vector and therefore are compatible with the illustrated FFNN. Encoder’s weights were set to be untrainable except for the weights of the added neural network. In essence, different types of encoders take in an uncompressed ECFP encoding (with a size of 8 × 512) and produce (output) a compressed representation (with a size 1 × 512) which is an input of a second (i.e., FFNN) performing label classification (task No. 1) or prediction of the ratio between solvents (task No. 2). The only difference in the ratio prediction task is that the output layer size is set to 1 neuron (instead of 20) because it corresponds to a single numeric value; also, a linear activation function is used instead of a sigmoid. The encoders were kept stable in this stage, while different topologies of the FFNN were investigated. These investigations involved the addition/removal of fully connected layers with different sizes equal to 16, 32, 64, 128, 256, 512. The determined optimal topology (able to work well with all types of encoders) is illustrated in Figure A4 (Appendix A): it contains three fully connected layers.

### 5.2. Supervised Machine-Learning Approach

In recent years deep learning has become the most popular approach from the supervised machine-learning group. DNNs often outperform classic supervised approaches and even can boost the accuracy to a higher level. DNNs are general approximators, they do not bind to specific fields and, therefore, are suitable for many applications, as well as for our tasks, i.e., multilabel classification (task No. 1) and regression (task No. 2) described in Section 3. In general, modeling the various chemical phenomena is difficult mostly due to large datasets that arise from the combinatorial nature of molecules and complex physicochemical relations between them. This is one more reason the most cutting-edge AI technologies in the field of chemistry rely on deep learning, using its ability to work with very large quantities of data. Thus, deep learning greatly benefits from big and diverse datasets (especially if relations between the input and output data are rather complex); therefore, it must be suitable for our datasets (Section 4) as well. In addition to large quantities and a variety of data, enough attention must be paid to the correct choice of the DNN method modification (type, topology, hyperparameter values), because it directly affects the accuracy.

The multiple specialized modifications (types) have been successfully developed for neural networks. Due to it, our focus is on the following ones that could be interesting and suitable for our solving tasks:

- Feed-Forward Neural Network (FFNN) is a simple DNN type in which information moves from input to output nodes without any loops. Despite it being much simpler than its successors, FFNNs have been successfully used to explore and visualize chemical space [48]. However, FFNNs are adjusted to learn the relationships between independent variables, which theoretically makes them unsuitable for solving tasks. Despite it, we will use FFNN in our experiments as the baseline approach to be able to compare the results with more sophisticated topologies.
- Convolutional neural networks (CNNs) [49] are a type of deep learning network initially developed for image recognition to detect recurring spatial patterns in the data. Recently CNNs are used in many applications to cope with the data, with grid patterns. Graph CNNs have been applied for predicting drug-target interactions [50]. Widely used CNN architectures VGG-19, ResNet512, AlexNet, DenseNet-201 [51,52,53,54] were used for the prediction of cytotoxicity for 8 cancer cell lines. In addition, CNNs have been proven to effectively identify steroids via deep learning retention time modeling when analyzed using gas chromatography [55]. It is hypothesized that molecules with similar chemical substructures or functional groups can behave similarly and form patterns recognized and processed by convolutional layers. It explains why CNNs are suitable for our solving tasks as well. Since we are dealing with sequences of symbols, we deal with models with 1D convolutions.
- Long short-term memory (LSTM) [56] neural networks are suitable for training on sequences due to a memory cell and feedback connections between cells. Contrary to their predecessors Simple Recurrent Neural Networks (RNNs), LSTMs do not suffer from the vanishing gradient problem and; therefore, learning longer sequential dependencies is not challenging anymore. Additional internal mechanisms (so-called gates) in LSTMs control information flow and carry relevant information forward while processing the sequence. Carrying information from earlier time moments in a sequence bears significant meaning on a conceptual level for the interpretation of chemical symbol sequences. For example, O and H symbols separately are less indicative than compared to (OH), which symbolizes a hydroxyl group or symbols c1 of aromaticity notation compared to an entire sequence cc1ccccc1 where c1 denotes a start and end of an aromatic ring. Thus, the sequential character of the input data explains the selection of LSTMs for our experiments.

Next to the DNN type, hyper-parameters (controlling the neural network learning process) play the very important role as well, therefore must be discussed:

- Activation functions. All layers except for the output one use rectified linear units (ReLU) activation function, and it is done on purpose. This activation function has statistically outperformed sigmoid and Tanh activation functions in similar bioactivity modeling tasks [57]. Additionally, ReLU is faster to compute compared to non-linear activation functions. Moreover, it generalizes better and converges faster [58]. In our preliminary tests, the ReLU activation function led to more stable training and convergence; moreover, it outperformed the other tested functions: i.e., Tanh, sigmoid, SoftMax, SeLU.
  The last layer’s activation function type fully depends on the solving task. Prediction of solvents is a multilabel prediction problem where the target output contains multiple independent and binary variables. We have used a binary cross-entropy loss function that works best with a sigmoid activation function. For task No. 1, we have chosen the sigmoid activation function because (1) it is the only compatible function with binary cross-entropy loss function used for loss calculation (2) sigmoid function’s returns values from the range [0–1]. Therefore, it is easier for the network to classify each binary label. As for task No. 2 (which is used to predict the ratio), the linear activation function is selected. This function is the most common activation function used for regression problems because it returns an unbounded numerical value.
- Optimizers. Optimizer Adam is a popular algorithm due to its ability to adjust learning rate according to circumstances in contrast to classical algorithms such as stochastic gradient descent (SGD), maintaining a single learning rate during the whole training process. In our experiments, Adam was selected due to these reasons: (1) the convergence of the model is significantly faster compared to classical optimizers; (2) the learning rate is controlled and does not lead to volatile training, especially at the end.
- Batch size. The batch size is one more important hyperparameter that impacts training stability and speed. Since the datasets (in Section 4) we are using in our research cover chemical experimental data collected in a long period of time, the factor of the noise in the data cannot completely be ruled out. Typically, to avoid a volatile learning process, a larger batch size is chosen to smooth over noisy instances. On the other hand, larger batch sizes require more V-RAM and slow down the learning process. Out of several options, 256 was selected to be a good compromise for our case. as the most effective.
- Epochs. During one epoch, the entire training dataset is passed through the network once. Since the learning process in DNNs is iterative, updating weights with only one pass is not enough. To avoid the negative effect of overfitting, the training dataset was split (90% training and 10% validation), and models were trained on 40 epochs: this number allowed to equalize the performance of training and validation loss functions during the model training process.

Optimization. Parameter (architecture and hyperparameter) optimization has been done manually by testing different network topologies and hyperparameter values. Activation functions (tanh, ReLU, SeLU), optimizers (Adam, SGD), batch size and training epoch count was varied while layers of different sizes (16, 32, 64, 128, 256, 512) were added or removed depending on whether it increased the model performance. The metrics of the validation dataset were monitored to judge the model performance, accuracy was monitored for task 1, R2 score for task 2.

**Figure A5:**
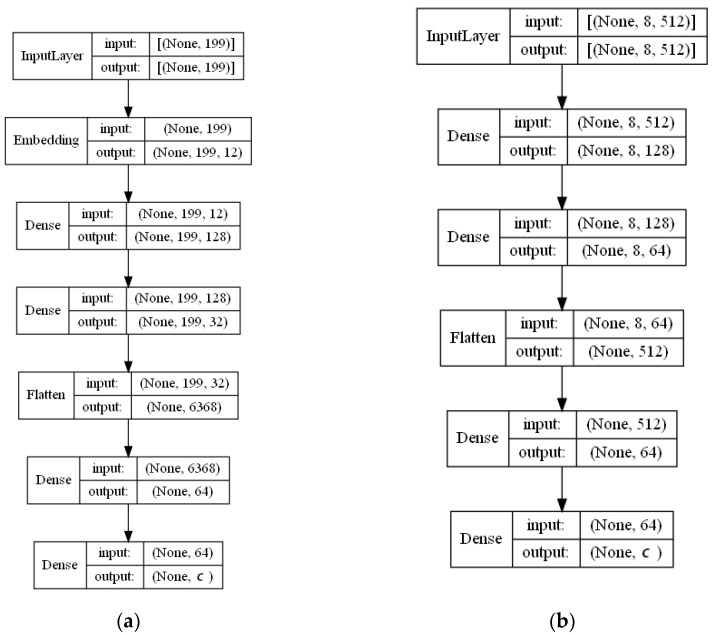
The topology of feed-forward neural network. Used with LE vectorization (**a**), with ECFP vectorization (**b**).

**Figure A6:**
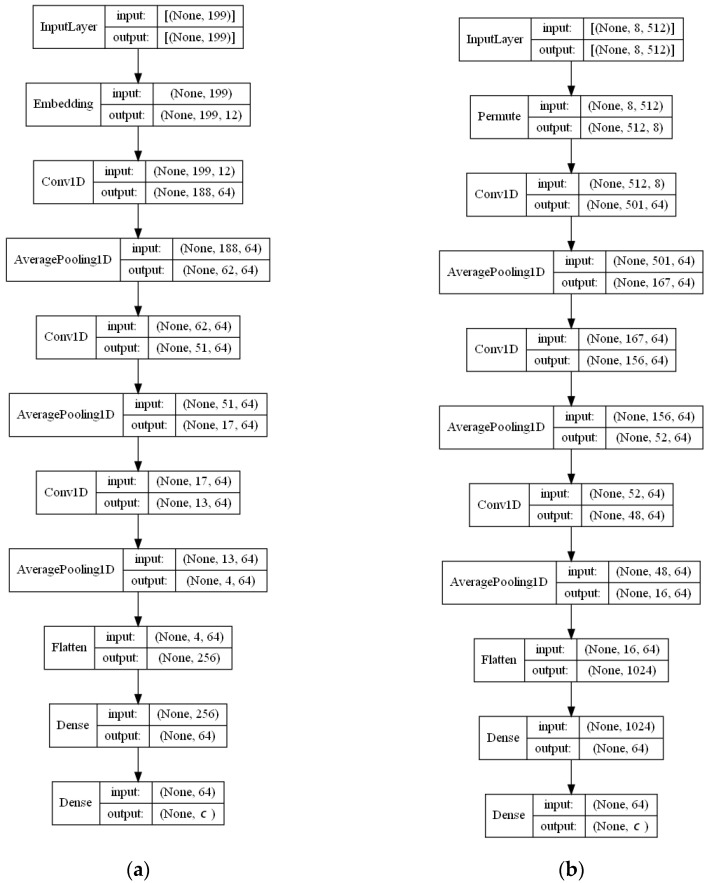
The 1D CNN neural network topology. Used with LE vectorization (**a**), with ECFP vectorization (**b**).

**Figure A7:**
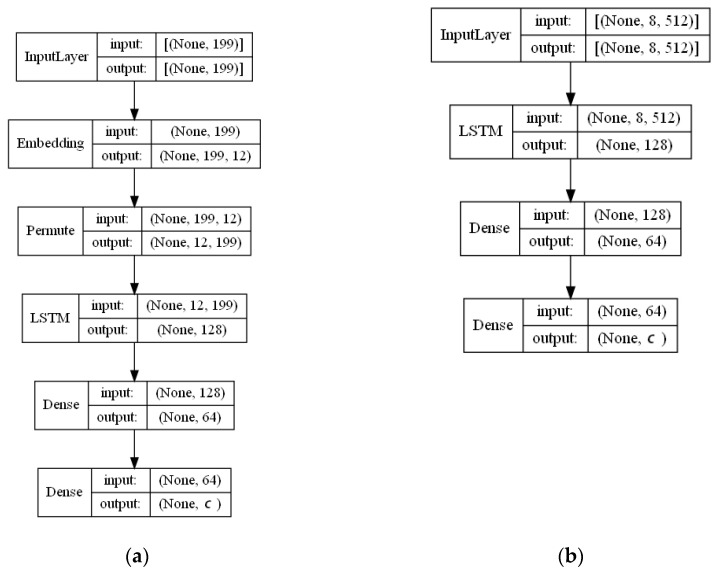
LSTM neural network topology. Used with LE vectorization (**a**), with ECFP vectorization (**b**).

Figure A5, Figure A6 and Figure A7 (Appendix A) illustrate topologies of DNNs used for the prediction of solvent labels (task No. 1) and the ratio between solvents (task No. 2). Topologies have been selected based on our own preliminary experiments, manual optimization, and guidelines from other similar works. Due to differences in the input format (i.e., used LE and ECFP vectorization), each figure illustrates two topologies for the same type of DNN. Various FFNN topologies have been constructed by adding and removing layers of different sizes (16, 32, 64, 128, 256, 512). 1D CNN has been tested by adding and removing convolutional layers of sizes (8, 16, 32, 64, 128, 256) with kernel sizes (2 to 20) and pooling layers of sizes (2, 3, 4, 5). LSTM neural network topology was developed by adding and removing layers with different sizes (16, 32, 64, 128, 256, 512). Figure A5, Figure A6 and Figure A7 (Appendix A) illustrate FFNN, CNN, and LSTM neural networks’ topologies. The neural network used with LE vectorization is displayed on the left side, with ECFP vectorization on the right. For the prediction of solvent labels (task No. 1) output layer’s size was set to 10 (variable denoted as c), for the prediction of the ratio between solvents (task No. 2) output layer’s size was set to 1 (variable c). Figure A5 (Appendix A) represents the topology of feed-forward neural network. Figure A6 (Appendix A) represents 1D CNN neural network topology. The determined optimal topology has three convolutional layers of sizes (64, 64, 64 in sequence with kernel sizes of 12, 12, (5) and average pooling layers of sizes (3, 3, 3). The activation function of all convolutional layers is ReLU. Figure A7 (Appendix A) represents LSTM neural network topology.

## 6. Results

The following experiments were performed on the datasets described in Section 4, using vectorization in Section 5.1 and methods in Section 5.2. The method architectures were implemented using python programming language and TensorFlow Keras API library [59]. For the ECFP vectorization RDKit library [60] was used.

### 6.1. Task No. 1. Prediction of Solvents Used in Chromatographic Purification (Section 3)

To interpret and compare obtained results, we need to determine evaluation metrics. For our multilabel classification task, accuracy (Equation (5)), precision (Equation (6)), recall (Equation (7)), and f-score (Equation (8)) were selected, based on similar works [61,62]. Notation: *TP* (true positives) determine cases when *Y_i_* was predicted as *Y_i_*, where *Y_i_* − 1, *Y_j_* − 0; *TN* (true negatives)—cases when *Y_j_* predicted as *Y_j_* (with *Y_j_* ≠ *Y_i_*); *FP* (false positives)—incorrect cases when *Yi* was predicted; *FN* (false negatives)—incorrect cases when *Y_j_* was predicted.

(5) $$\mathrm{Accuracy}=\frac{TP+TN}{TP+FP+TN+FN}$$

(6) $$\mathrm{Precision}=\frac{TP}{TP+FP}$$

(7) $$\mathrm{Recall}=\frac{TP}{TP+FN}$$

(8) $$F1\;\mathrm{score}=\frac{2\times precision\times recall}{precision+recall}$$

**Figure 2:**
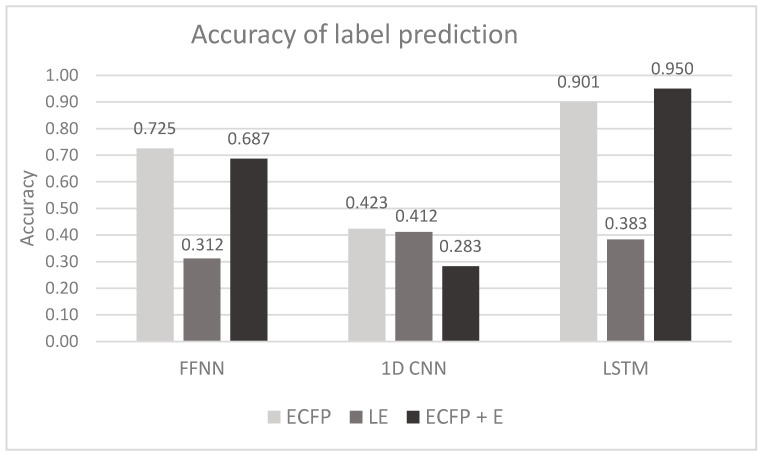
Accuracies with different vectorization types and methods for task No. 1. The majority baseline is equal to 0.313.

Experiments were performed to test three vectorization methods (ECFP, LE, ECFP + E (Section 5.1)) with three DNN types (FFNN, 1D CNN, LSTM (Section 5.1)) (see Table 5). The evaluation of each combination was averaged in 3 runs (each time training and testing on the corresponding training and testing datasets), and the confidence intervals were calculated (with the confidence level = 95%). Figure 2 visually presents the obtained results (accuracies).

### 6.2. Task No. 2: Prediction of Ratio between Solvents, When Their Number Is Two

The prediction of the ratio between solvents is a regression problem (Section 3). Therefore, for neural networks that predict ratio, we used the default loss function—mean squared error (MSE). MSE is superior since the result is positive regardless of the predicted and true values. Moreover, because the difference between the predicted and true value is squared, bigger prediction mistakes result in significantly higher loss and lead to more stable model training and faster convergence.

For the regression results evaluation, multiple metrics were used R-squared (Equation (9)), Pearson product-moment correlation (Equation (10)) and MSE (Equation (11)). Notation: $y_{i}$*,*$\;\hat{y_{i}}$—real and predicted values, $\bar{y}\;$—mean of real values,$\;\bar{x}\;$—mean of predicted values, $\sigma_{y},\;\sigma_{x}$—standard deviation of real and predicted values, *n*—object count.

(9) $$\mathrm{R-squared}=1-\frac{\sum_{i=1}^{n}{\left(y_{i}-\hat{y}\right)}^{2}}{\sum_{i=1}^{n}{\left(y_{i}-\bar{\;y}\right)}^{2}}$$

(10) $$\mathrm{Pearson}\;R=\frac{1}{n}\sum_{i=1}^{n}\frac{\left(y_{i}-\bar{y}\right)}{\sigma_{y}}\times \frac{\left({\hat{y}}_{i}-\bar{x}\right)}{\sigma_{x}}$$

(11) $$\mathrm{MSE}=\frac{1}{n}\sum_{i=1}^{n}{\left(y_{i}-\hat{y}\right)}^{2}$$

**Figure 3:**
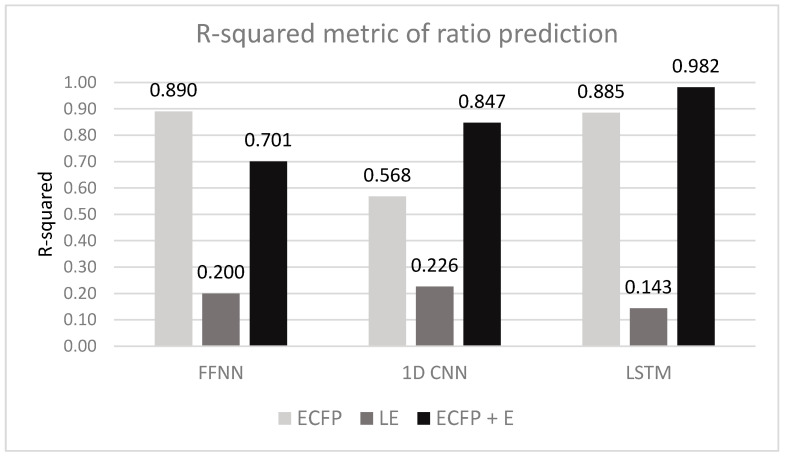
Pearson R values for task No. 2.

Task No. 2 entails predicting a numeric value that represents the ratio in a two-solvent system. Three vectorization methods (ECFP, LE, ECFP + E (Section 5.1)) and (FFNN, 1D CNN, LSTM (Section 5.2)) were tested and evaluated. Table 6 presents metrics between ground truth values and predicted values. Figure 3 visually displays the obtained results.

## 7. Discussion

Because the task is focused on a wide variety of molecules and solvent systems for which we use a unique dataset, we are unfortunately unable to compare the results of this paper to previous studies directly. To establish a baseline using traditional approaches, we have performed tests using Naive Bayes classifier for task 1, resulting in an accuracy of 0.143 ± 0.09 and a Linear Regressor for task 2, which resulted in R^2^ score of 0.260 ± 0.07.

Zooming into the results in Table 5 and Figure 2 of task No. 1 allows us to make the following statements. 2 of 9 tested methods (in particular, LE + FFNN and ECFP + E (CNN)) cannot exceed the majority baseline; therefore, they are considered unsuitable for our task. The LE vectorization with FFNN is likely not complex enough to fully capture relations between input and output data since FFNN does not have feedback loops. When looking at ECFP + E (CNN) vectorization, poor results may arise from the fact that an auto-encoder constructed with convolutional layers cannot learn meaningful patterns because input data is too sparse. In general, CNNs perform poorly with other types of vectorization, and ECFP + E (CNN) vectorization is not an exception.

As for vectorization types, learned embedding achieves the lowest accuracy scores with all types of tested DNNs (0.412). The LE vectorization, in theory, should produce similar results seen with an encoder; however, for learned embedding vectorization, the primary input is a sequence of SMILES symbols denoting chemical compounds, which possibly are less informative than fingerprints. Learned embedding encodes each symbol as a multi-dimensional vector; however, compared to ECFP it encodes individual symbols, not groups, which might be the reason for low performance. The most significant feature for the selection of solvents used for liquid chromatography is functional groups of molecules that are composed of multiple atoms, for example –OH, -COH. Vectorization using ECFPs provides a way to capture and encode such groups’ presence or absence. A reasonable assumption would be that a machine-learning algorithm could model a solvent system for a set of reactants when given molecules’ chemical features. The statement is supported by the fact that the use of ECFPs results in higher accuracy when compared to learned embedding.

**Figure 4:**
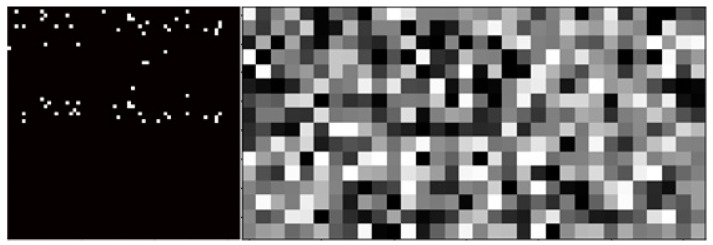
Visualization of vectorized reaction representations. ECFP (left), ECFP + E (LSTM) (right).

It is reasonable that Morgan Fingerprints and encoders produce similar results despite the supervised machine-learning method. First, Morgan Fingerprints are calculated and later compressed with an encoder (more details in Section 5.1). Based on experimental results, it can be concluded that encoders compress Morgan Fingerprints in a meaningful way to produce a less sparse representation of multiple compounds of a reaction. A condensed representation allows neural networks to learn patterns easier when compared to raw Morgan Fingerprints. Figure 4 presents vectorized inputs visually; input matrices are reshaped for simpler visualization. Each square depicts a single number of the input; lower values are black, higher are white. ECFPs (left) are quite sparse with a total size of 4096, and the encoder’s output (right) is a compressed version of ECFPs with a total size of 512. It is apparent why it is easier for a neural network to learn patterns when an encoder is used since the compressed version does not have large non-zero segments.

Excluding the results with Learned embedding (lowest performance with any supervised machine-learning approach), the worst machine-learning method is CNN. Theoretically, CNNs should cope with similar inputs as in our case. A reasonable guess would be that the input data is too sparse; it prevents CNNs from learning meaningful patterns. It is noticeable that certain types of neural networks only work well with certain types of vectorization. In particular, ECFP and ECFP + E with LSTM neural networks. The most accurate predictions are produced by ECFP + E (LSTM) neural network type (0.950 ± 0.001), most likely because it benefits from the input compression by an encoder and feedback loops of the LSTM layer. The statement can be backed up by results of the 2nd most accurate configuration (ECFP + LSTM (0.901 ± 0.001)), which also features the LSTM layer; however, it works with uncompressed ECFPs as input and produces slightly lower scores.

Since in our experiments we have used the imbalanced dataset (Section 4), it was important to zoom into the results for different classes (summarized in Table 7 of the most accurate model (ECFP + E (LSTM))). Surprisingly, classes covered by fewer instances appear to be similarly predicted as the larger ones. It allows us to believe that even rarer classes have enough representative examples to be learned.

From task No. 2 experimental results in Table 6, it can be concluded that learned embedding vectorization type results in the least accurate predictions (R^2^ < 0.226) for all types of tested DNN types, compared to ECFP or ECFP + E vectorization. Similar to task No. 1, learned embedding, in contrast to ECFP, encodes symbols separately and results in less accurate predictions. In general, ECFP and ECFP + E vectorization performs reasonably well (R^2^ > 0.568); however, ECFP + E (LSTM) produces the most accurate predictions (R^2^ 0.982 ± 0.001), similar to task No. 1, where the same type of encoding and type of neural network produces most accurate predictions. It is reasonable that the same configuration results in both tasks’ highest scores. Results of all models are better than mean and median baseline metrics.

The best performing method is ECFP + E (LSTM); however, the gap between the other approaches producing similar results (e.g., ECFP + FFNN, ECFP + LSTM) is not that apparent as in the previous tasks. It is probably because task No. 2 is a less complex task when compared to task No. 1 because in this case, only one number must be predicted and different types of a neural network can deal reasonably with the task.

The idea behind those two separately solved tasks is to use them sequentially. The first model is responsible for the prediction of solvents. In the case of two predicted solvents, the second model predicting the ratio between solvents is activated. Based on the obtained results, for both models (classification and regression) the same ECFP + E (LSTM) method would be recommended. Also, a graph convolution neural network (GCN) that accepts a graph as input, where vertices represent individual atoms and edges represent bonds, has been tested for comparison. The modified neural networks have been shown to match or beat standard fingerprints’ predictive performance on solubility, drug efficacy datasets [63]. For our task, an accuracy score of 0.607 ± 0.04 has been reached for task 1 (solvent prediction) and R^2^ score of 0.711 ± 0.04 for task 2 (ratio prediction). However, both models underperform compared to models presented in this paper (ECFP + E (LSTM)). Despite the promising results are achieved in the artificial environment, it opens the gate for further testing it in a real laboratory environment. It is important to repeat these predictions in a real-life setting for several reasons. First, the data is collected from patents that range from 1976 to 2010; procedures of how the reactions are performed can influence what by-products could be contaminating the final mixtures that are purified. Moreover, even if procedures are similar, the human factor can affect how syntheses are performed and lead to additional deviation from the expected mixture composition. Secondly, it is unclear how well the model that is not bounded to specific compound or reaction types perform for a wide variety of possible combinations of molecules and target products. Thirdly, our dataset’s chromatographic purification conditions are inherently tied to the synthesis procedure because additional steps may be taken that precede the chromatographic purification step, such as extraction or crystallization. However, our research models are promising due to several reasons. First, are not trained for specific reaction type or a set of compounds and do could be used for various syntheses. Secondly, do not require compound structural knowledge of by-products that are in the mixture for the prediction of chromatographic purification. To make the argument more compelling, it would be meaningful to compare models trained on examples with multiple main products; however, only 1.86% of all reactions in the dataset result in two or more products, while the rest use column chromatography to purify impurities. A subset of reactions with multiple main products would be unreasonably small to compare with those presented in the paper. The original dataset does not contain enough examples for such comparison, and it would have to be expanded to include more examples with multiple reaction products. In addition, a model trained just on products has been evaluated. An auto-encoder maps the 2D into 1D vector of size 1 × 512. To test the model’s accuracy with just products, the input is already a 1D vector of size 1 × 512, and the most suitable type is ECFP vectorization. Vectorized input was tested with all classification approaches and resulted in accuracy of 0.384 ± 0.04, 0.379 ± 0.04, 0.338 ± 0.05 for FFNN, CNN, LSTM, respectively. As we can see, even the best model (with the highest achieved accuracy of 0.384 ± 0.04) underperforms compared to models trained on reactants and products. Moreover, compared to retention time modeling for chromatography, the solvent system is predicted directly and does not require additional tuning.

The use of auto-encoders for the compression of fingerprints on its own is interesting because similar setups could be used for other problems in chemical data modeling since molecular representation is a challenging issue. Researchers have explored various methods of vectorization, such as descriptors, graphs, SMILES vectorization, and other hybrid methods that combine multiple methods into a single vector. Further options for exploration of the developed auto-encoder may include the addition of descriptors, different types of fingerprints, or parameters of Morgan Fingerprints, and all it is in our future plans.

Solvents and temperature used in the synthesis reaction process may play a role in selecting solvents. We have investigated the dataset; unfortunately, only around 20% of reactions contain information about the solvent used in the primary stages of the procedure, when products forms, and the solvent may influence the impurities. Additionally, temperature and reactant solvent are both present in 7.3% of the data. It is very likely that the accuracy of the model for prediction of the purification conditions would be significantly lower because of a small dataset which would counteract benefits from additional features. In addition, a less diverse dataset would negatively affect the versatility of the model. Also, we have evaluated whether similar reactions in the dataset use different solvent systems. This has been done by analyzing reactions with the same products. In the dataset, 3077 reactions have a duplicate regarding the product; however, they use different reactants. To evaluate similarity, we have used Levenshtein’s distance to compare the SMILES strings of compounds that denote these structures. Reactions with lesser Levenshtein’s distance than 15 were considered similar, while others have been observed to contain significantly different molecular structures. Out of all 3077 reactions, 174 (5.7%) used different solvent systems. A probable cause for the difference between similar reactions may arise from the fact that other solvents or temperature settings have been used during the process of synthesis. Another important future direction could be an augmentation of models to be able to predict more solvent labels. Unfortunately, it cannot be done straightforwardly by including additional 8 labels from the source dataset. Those additional labels comprise less than 8% of the entire source dataset and would highly imbalance the one we are working with. Moreover, it is estimated that the dataset we are using for our experiments contains 2–4% of noisy instances with incorrect labels for solvents used in chromatographic purification. The manual correction would be a laborious task due to the dataset’s large size. Perhaps the current model could help to semi-automate the correction process. Such an idea is not without reason. We have noticed that some incorrect labels (considered to be the gold standard in our testing dataset) were predicted correctly during the manual error analysis. Such an example raises a reasonable doubt that the achieved results are even slightly higher than those reported in this paper.

## 8. Conclusions

In this research, we solve two novel tasks based on the prediction of chromatographic purification conditions, i.e., solvents and the ratio between solvents. By tackling these tasks, three vectorization types (Learned embedding, extended-connectivity fingerprints, ECFP encoder + FFNN), three supervised machine-learning approaches (FFNN, LSTM, CNN), various DNN architectures, and a set of hyperparameter values were investigated.

The best results on both prediction tasks (i.e., prediction of solvents used in chromatographic purification and prediction of the ratio between solvents) were achieved with extended-connectivity fingerprint LSTM auto-encoder with FFNN as the supervised machine-learning method. The first task reached an accuracy of 0.950; whereas the second produced R^2^ of 0.982.

The best prediction accuracy of solvents used in chromatographic purification exceeds random and majority baselines by a significant margin of 0.778 and 0.637, respectively. The best Pearson R-value (which is >0.7) for the prediction of the ratio between solvents indicates a strong linear relationship between predicted and real ratio values. These results allow us to claim that the models can be used as a guidance instrument in laboratories to accelerate scouting for suitable chromatography conditions. In the future we are planning: (1) to continue working with the sequential models (LSTMs, BiLSTMS, transformers) that were proved to be the most suitable for our solving tasks; (2) to expand the current dataset by including more solvent labels for more complex purification solvent systems and to cover an even larger variety of synthesis procedures; (3) to perform real-life laboratory testing to explore our model’s ability to predict chromatographic purification conditions accurately and assist researchers with suggestions. Unfortunately, they require a set of reagents, laboratory time, and financial and human resources. Since purification is done towards the end of reaction procedures, all necessary steps, such as preparation, synthesis, work-up must also be done. To reasonably compare traditional and our purification condition scouting method, a minimum of 150–250 reactions need to be done. This amounts to a considerable length of time and therefore experiments are planned for further research work.

## Figures and Tables

**Table 1:** An example of the extracted data from the *purification* step within the synthesis instruction. Regions in bold text draw attention to the exact parts that were extracted.

Raw Text	Solvents	Ratio
the residue is chromatographed on silica gel with **hexane/ethyl acetate 9:1**	*Hexane,* *ethyl acetate*	9:1
the residue was purified by column chromatography on silicagel with **ethylacetate/methanol (1:1)**	*Ethyl acetate, methanol*	1:1
the residue was chromatographed rapidly on 300 g of silica gel 60 eluting with ethyl **acetate/hexane** (**2:3** parts by volume)	*Hexane,* *ethyl acetate*	2:3

**Table 2:** The table illustrates examples from DS1 (the first one) and DS2 (the second one) before the vectorization (*d_i_*).

Dataset 1 (DS1)	
**Input**	**Output (Labels)**
OCc(cc1)cc2c1OCO2.BrCC = CCBr.[Na].BrCC = CCOCc(cc1)c1OCO2	Hexane, ethyl acetate
**Dataset 2 (DS2)**	
**Input**	**Output (Ratio)**
OCc(cc1)cc2c1OCO2.BrCC = CCBr.[Na].BrCC = CCOCc(cc1)c1OCO2	2:3

**Table 3:** Distribution of instances over different class labels (DS1).

Class Label	Training Subset (Number of Instances)	Testing Subset (Number of Instances)	Total (Number of Instances)
Ethyl acetate	255,251	28,361	283,612
Hexane	155,858	17,318	173,175
Dichloromethane	85,643	9516	95,159
Methanol	84,626	9403	94,029
Chloroform	31,582	3509	35,091
Petroleum ether	16,964	1885	18,849
Diethyl ether	7943	883	8825
Toluene	7636	848	8484
Acetone	6139	682	6821
Ethanol	3424	380	3804

**Table 4:** Descriptive statistics about dataset DS2.

Dataset 2			
	Training Subset	Testing Subset	Total
Numb. of instances	245,096	27,233	272,329
Mean	0.356	0.355	0.356
Standard deviation	0.306	0.304	0.306
Minimum value	0.001	0.001	0.001
25% quartile	0.100	0.100	0.100
50% quartile	0.250	0.250	0.250
75% quartile	0.500	0.500	0.500
Maximum value	0.998	0.998	0.998

**Table 5:** Evaluation results of task No. 1.

Vectorization	Training Dataset	FFNN	1D CNN	LSTM
ECFP	Accuracy Precision Recall F1-score	0.725 ± 0.002	0.423 ± 0.002	0.901 ± 0.001
		0.899 ± 0.002	0.686 ± 0.004	0.960 ± 0.002
		0.862 ± 0.003	0.709 ± 0.004	0.962 ± 0.001
		0.697 ± 0.003	0.880 ± 0.004	0.961 ± 0.002
LE	Accuracy Precision Recall F1-score	0.312 ± 0.005	0.412 ± 0.012	0.383 ± 0.019
		0.605 ± 0.004	0.687 ± 0.004	0.628 ± 0.007
		0.603 ± 0.006	0.708 ± 0.003	0.683 ± 0.004
		0.654 ± 0.005	0.604 ± 0.004	0.654 ± 0.005
ECFP + E	Accuracy Precision Recall F1-score	0.687 ± 0.002	0.283 ± 0.002	0.950 ± 0.001
		0.845 ± 0.004	0.558 ± 0.004	0.977 ± 0.002
		0.807 ± 0.006	0.591 ± 0.011	0.979 ± 0.002
		0.574 ± 0.005	0.825 ± 0.007	0.978 ± 0.002

**Table 6:** Evaluation results of task No. 2.

Vectorization	Training Dataset	FFNN	1D CNN	LSTM
ECFP	R-squared Pearson R MSE	0.890 ± 0.001	0.568 ± 0.002	0.885 ± 0.001
		0.941 ± 0.005	0.691 ± 0.001	0.941 ± 0.001
		0.013 ± 0.001	0.040 ± 0.001	0.010 ± 0.001
LE	R-squared Pearson R MSE	0.200 ± 0.002	0.226 ± 0.004	0.143 ± 0.003
		0.459 ± 0.001	0.475 ± 0.004	0.372 ± 0.002
		0.080 ± 0.001	0.072 ± 0.001	0.084 ± 0.003
ECFP + E	R-squared Pearson R MSE	0.701 ± 0.003	0.847 ± 0.002	0.982 ± 0.001
		0.831 ± 0.003	0.896 ± 0.007	0.984 ± 0.001
		0.026 ± 0.002	0.013 ± 0.001	0.002 ± 0.001

**Table 7:** Results for separate classes with the best performing method ECFP + E (LSTM).

Class Label	Precision	Ratio	F1-Score	Number of Examples
Chloroform	0.976	0.955	0.966	3424
Dichloromethane	0.980	0.956	0.968	9626
Ethyl acetate	0.983	0.990	0.986	28,472
Diethyl ether	0.921	0.951	0.936	834
Hexane	0.969	0.979	0.974	17,216
Petroleum ether	0.956	0.947	0.952	1836
Acetone	0.964	0.939	0.951	680
Toluene	0.974	0.935	0.954	817
Methanol	0.979	0.964	0.972	9558
Ethanol	0.949	0.967	0.958	354

## Data Availability

Publicly available original dataset can be found: https://figshare.com/articles/dataset/Chemical_reactions_from_US_patents_1976-Sep2016_/5104873 (accessed on 5 December 2020). The extracted datasets and code used in this paper can be found: https://github.com/Mantas-it/Chrom_cond_pred (accessed on 12 March 2021).
